# Recent Findings in N6‐Methyladenosine Modification and Significance in Pancreatic Cancer

**DOI:** 10.1002/cam4.70934

**Published:** 2025-05-30

**Authors:** Tomoaki Hara, Sikun Meng, Yasuko Arao, Yoshiko Saito, Kana Inoue, Sarah Rennie, Ken Ofusa, Toru Kitagawa, Hideshi Ishii

**Affiliations:** ^1^ Department of Medical Data Science, Center of Medical Innovation and Translational Research Osaka University Graduate School of Medicine Osaka Japan; ^2^ Section for Computational and RNA Biology, Department of Biology University of Copenhagen Copenhagen Denmark; ^3^ Prophoenix Division, Food and Life‐Science Laboratory IDEA Consultants, Inc. Osaka Osaka Japan; ^4^ Kyowa‐Kai Medical Corporation Kawanishi Hyogo and Osaka Japan

**Keywords:** m^6^A, METTL3, pancreatic cancer, RNA modifications, TCEAL8

## Abstract

**Background:**

RNA modifications are widely detected in cells and are involved in RNA structural stabilization and regulation of gene expression. In cancer cells, RNA modifications are altered, resulting in abnormal expression of numerous genes and promoting cancer growth. N1‐methyladenosine (m^1^A), N6‐methyladenosine (m^6^A), N3‐methylcytosine (m^3^C), 5‐methylcytosine (m^5^C), 7‐methylguanosine (m^7^G), and N4‐acetylcytidine (ac^4^C) have been reported as RNA modifications affecting gene expression.

**Aim:**

In this review, the function of m^6^A in pancreatic cancer is mainly described, and the current status and prospects of RNA modifications are discussed.

**Methodology:**

We summarize recent reports on m^6^A writers METTL3, METTL5, METTL14, and METTL16; m^6^A readers IGF2BP1, IGF2BP2, IGF2BP3, YTHDF1, YTHDF2, and YTHDF3; and m^6^A erasers ALKBH5 and FTO.

**Results:**

RNA modifications are written to the RNA by the writer, and the reader binds to the RNA modification, causing gene expression to increase or decrease. Gene expression is also regulated by the removal of RNA modifications by the eraser. Moreover, our recent investigation into m^6^A modifications in pancreatic cancer has led to the identification of several promising candidate biomarkers, highlighting the potential role of epitranscriptomic regulation in tumorigenesis.

**Conclusion:**

These findings suggest that further exploration of RNA modification functions may facilitate the identification of novel biomarker and therapeutic target molecules for pancreatic cancer.

## 1 Background

**FIGURE 1:**
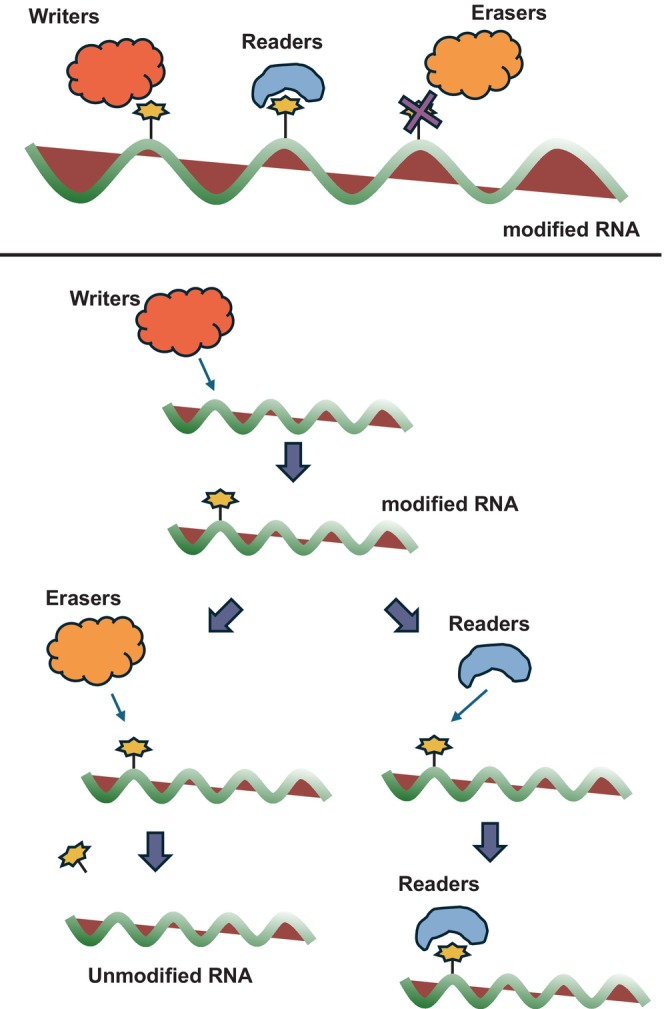
Writers, readers, and erasers in RNA modification. Writers perform modifications to RNA, Readers recognize and bind the modified RNA, and Erasers remove RNA modifications.

Pancreatic cancer is difficult to detect in its early stages and is one of the most difficult‐to‐treat cancers with a low 5‐year survival rate [1]. Therefore, there is an urgent need to develop diagnostic methods for early detection and new medicines for treatment. For this purpose, marker molecules and target molecules of therapeutic medicines for pancreatic cancer have been explored [2, 3]. These have been identified by analyzing mutated genes, gene expression patterns, and molecules in the exosome found in pancreatic cancer [4, 5]. Among them, analysis focusing on RNA modifications has attracted much attention [6]. RNA modifications are mainly found in ribosomal RNA (rRNA) and transfer RNA (tRNA), but RNA modifications have been found to occur in messenger (mRNA) as well [7]. Adenine modifications in HeLa cell and HEK293 cell mRNA were reported to be about 0.04% for m1A and 0.5% for m^6^A, and m^1^A modifications were mostly found in the 5′ untranslated region (5′ UTR) near the start codon, indicating that they are involved in translation regulation [8]. In addition, about 7000 m^5^C and 6900 ac^4^C modifications were found in HeLa cell mRNA, and gene ontology (GO) enrichment analysis revealed that m^5^C and ac^4^C modifications were found in mRNA of genes related to cadherin binding and ubiqutin protein ligase binding [9]. In HCT116 cells, approximately 0.004% of cytosine residues in mRNA were modified with m^3^C, whereas around 0.009% were modified with m^5^C, and about 0.006% of adenine residues in mRNA carried m^1^A modifications, whereas approximately 0.15% were modified with m^6^A [10]. Furthermore, about 0.2% of uridine in HEK293T cell mRNA was pseudouridine [11]. The m^7^G modification is essential for mRNA cap structure and is involved in the translation of all mRNAs [12]. Regulation of translation efficiency and splicing by m^6^A modifications may affect gene expression. Thus, modifications have been detected in mRNA, in particular, m^6^A has been detected in many genes and has been found to have a significant effect on gene expression [13]. m^6^A is added to RNA molecules by writer‐mediated RNA modification, binding by readers inhibits or promotes RNA degradation, and erasers removes m^6^A and regulates gene expression [14] (Figure 1). In this review, we summarize the recently discovered function of m^6^A in pancreatic cancer, including our recent studies.

## 2 Writers

### 2.1 METTL3

In this section, we present seven cases recently reported as target RNAs of methyltransferase 3 (METTL3). DNA cross‐link repair 1B (DCLRE1B), a 5′‐to‐3′ exonuclease, a DNA repair gene, is upregulated in pancreatic cancer, promotes the proliferation of pancreatic cancer cells, and is associated with poor prognosis. DCLRE1B expression activates the JAK–STAT signaling pathway, promotes immune checkpoint (ICP) gene expression, and influences immunotherapy sensitivity. DCLRE1B mRNA is stabilized and upregulated after m^6^A modification by METTL3 [15]. lncRNA NNT‐AS1 is highly expressed in pancreatic cancer by HIF‐1α‐mediated transcription, and m^6^A‐modified NNT‐AS1 by METTL3‐HuR stabilizes ITGB1. Knockdown of NNT‐AS1 reduced ITGB1 expression and growth of pancreatic cancer cell lines, but overexpression of ITGB1 restored growth of pancreatic cancer cell lines even when NNT‐AS1 was knocked down. In addition, knockdown of NNT‐AS1 in a tumor xenograft mouse model suppressed cancer growth [16]. Amphoteric regulatory protein (AREG), a member of the epidermal growth factor (EGF) family, is upregulated in pancreatic cancer. AREG mRNA is stabilized by m^6^A modification by METTL3, and the upregulation of AREG expression promotes cancer progression. miR‐33a‐3p suppresses METTL3 expression and decreases m^6^A modification of AREG mRNA [17]. METTL3 is upregulated in pancreatic cancer, and knockdown of METTL3 in a mouse xenograft model suppressed cancer growth. E2F transcription factor 5 (E2F5) mRNA is m^6^A‐modified by METTL3, and E2F5 expression is upregulated. Overexpression of E2F5, even when METTL3 is knocked down, could promote cancer growth [18]. lncRNA LIFR‐AS1 is stabilized by m^6^A modification by METTL3 and promotes pancreatic cancer growth. LIFR‐AS1 sponges miR‐150‐5p and upregulates the expression of Vascular Endothelial Growth Factor A (VEGFA), a target of miR‐150‐5p. Knockdown of LIFR‐AS1 reduced VEGFA expression and inhibited AKT and mTOR phosphorylation, suggesting that LIFR‐AS1 contributes to the activation of AKT/mTOR Signaling [19]. Smoking induces hypomethylation of the promoter region of METTL3, activating METTL3 transcription and upregulating METTL3 expression. METTL3 stabilizes pri‐miR‐25 by modifying it with m^6^A, resulting in increased expression of miR‐25‐3p and decreased expression of PHLPP2, a target of miR‐25‐3p. When PHLPP2, a PH domain leucine‐rich repeat protein phosphatase 2, was knocked down, an increase in phosphorylation of AKT was observed, suggesting that m^6^A modification of pri‐miR‐25 ultimately contributes to the activation of the AKT pathway and pancreatic cancer progression [20]. Expression of lncRNA DBH‐AS1 is suppressed in gemcitabine‐resistant pancreatic cancer. DBH‐AS1 is upregulated by m^6^A modification by METTL3 and sponges miR‐3163, resulting in the upregulation of Ubiquitin Specific Peptidase 44 (USP44), a target gene of miR‐3163. In the xenograft mouse model, overexpression of USP44 inhibited tumor growth, even when DBH‐AS1 was knocked down. In patient‐derived xenograft (PDX) models with high or low expression of DBH‐AS1, when cancer growth in the presence of gemcitabine was examined, the growth rate was higher in the group with low expression of DBH‐AS1, suggesting that DBH‐AS1 is associated with USP44‐mediated cancer growth and gemcitabine resistance. DBH‐AS1 is upregulated by METTL3‐mediated m^6^A modification, and overexpression of DBH‐AS1 sponges miR‐3163 and upregulates the expression of USP44, a target gene of miR‐3163, thereby reducing gemcitabine resistance and suppressing cancer growth [21]. It has been interestingly reported that lactate upregulates METTL3 expression in colon cancer. Lactate accumulated in the colon cancer microenvironment is taken up by tumor‐infiltrating myeloid cells (TIMs) and histone H3K18 lactylation activates METTL3 transcription, resulting in increased METTL3 expression. The zinc‐finger domain of METTL3 contains two lactylation sites, and lactylation enhances binding to the METTL3 target RNA. JAK1 mRNA is modified by m^6^A and stabilized by binding to YTHDF1, and upregulation of JAK1 enhances the JAK1/STAT3 signaling pathway and suppresses immunity by upregulating interleukin 6 (IL‐6) and interleukin 10 (IL‐10) expression [22]. A similar mechanism may exist in pancreatic cancer. As described above, our knowledge of RNAs modified by METTL3 and their functions is accumulating. It appears that m^6^A modification of METTL3 increases gene expression and enhances proliferative signals in cancer.

### 2.2 METTL5, METTL14, and METTL16

**FIGURE 2:**
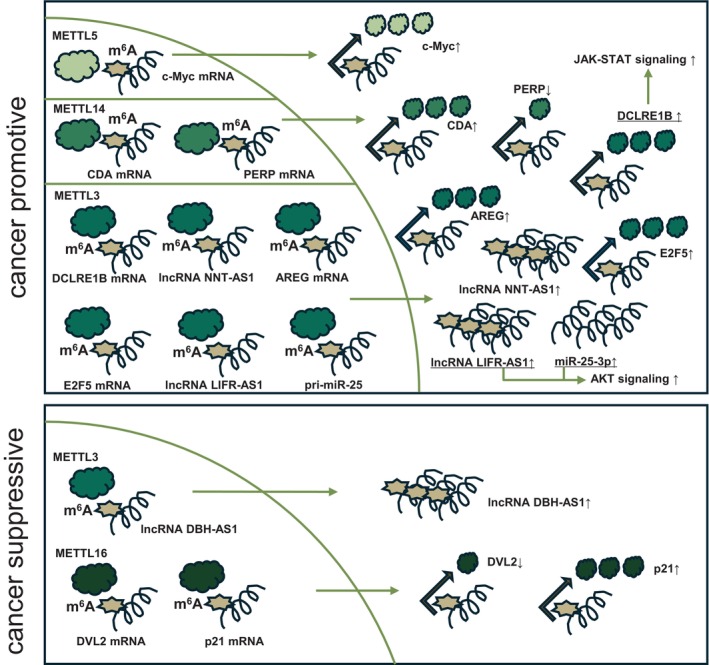
Regulation of gene expression by writers. mRNA is m^6^A‐modified in the nucleus and mRNA is translationally regulated in the cytoplasm, whereas ncRNA is involved in translational regulation.

In addition to METTL3, there are other molecules that regulate gene expression through m^6^A modifications. In this section, we describe five examples recently reported. Methyltransferase 5 (METTL5) catalyzes 18S rRNA N6‐methylation at adenosine 1832 (m^6^A1832) and overexpression of METTL5 in a mouse xenograft model resulted in tumor growth. Although c‐Myc mRNA is m^6^A modified and c‐Myc expression levels were found to be slightly positively correlated with METTL5, further studies are needed to confirm whether c‐Myc mRNA is a direct target of METTL5 [23]. Methyltransferase 14 (METTL14) is upregulated by gemcitabine treatment, and knockdown of METTL14 confirmed increased sensitivity to gemcitabine. Mechanistically, METTL14 is transcriptionally activated by p65 and increases gemcitabine resistance by upregulating expression of cytidine deaminase (CDA), a gemcitabine‐inactivating enzyme [24]. Furthermore, overexpression of METTL14 promoted pancreatic cancer growth. The mRNA of p53 apoptosis effector related to PMP22 (PERP) involved in apoptosis, was found to be destabilized and down‐regulated upon m^6^A modification by METTL14 [25]. Methyltransferase 16 (METTL16) is downregulated in pancreatic cancer, and its knockdown promotes metastasis and invasion. Although METTL16 destabilizes disheveled segment polarity protein 2 (DVL2) mRNA by m^6^A modification and suppresses its expression, when the expression of DVL2 increases due to decreased METTL16 expression, Wnt/β‐catenin signaling is activated and cancer progression occurs [26]. It was also observed that overexpression of METTL16 inhibited cancer growth. Mechanistically, METTL16‐mediated m^6^A modification of p21 mRNA upregulates p21 expression and suppresses cyclin dependent kinase 1 (CDK1)/Cyclin B, thereby inhibiting cell proliferation [27]. Thus, the expression of various genes is regulated by writers (Figure 2 and Table 1). METTL family has been identified as both cancer‐promoting and cancer‐suppressing. Although METTL3 has been the main focus of research so far, further functional analysis of other METTL family members will be increasingly needed in cancer research.

**TABLE 1:** Targets of writers and their functions in pancreatic cancer.

Writers	Targets	Expression	Modification effect on tumor	Experiments	References
METTL3	DCLRE1B	Up	Promotive	Cell lines	[15]
METTL3	lncRNA NNT‐AS1	Up	Promotive	Cell lines and mouse	[16]
METTL3	AREG	Up	Promotive	Cell lines and human PC tissues	[17]
METTL3	E2F5	Up	Promotive	Cell lines and mouse	[18]
METTL3	lncRNA LIFR‐AS1	Up	Promotive	Cell lines	[19]
METTL3	pri‐miR‐25	Up	Promotive	Cell lines and human PC tissues	[20]
METTL3	lncRNA DBH‐AS1	Up	Suppressive	Cell lines and mouse	[21]
METTL5	c‐Myc	Up	Promotive	Cell lines and mouse	[23]
METTL14	CDA	Up	Promotive	Cell lines and mouse	[24]
METTL14	PERP	Down	Promotive	Cell lines, mouse, and human PC tissues	[25]
METTL16	DVL2	Down	Suppressive	Cell lines and human PC tissues	[26]
METTL16	p21	Up	Suppressive	Cell lines, mouse, and human PC tissues	[27]

Abbreviation: PC, pancreatic cancer.

## 3 Readers

### 3.1 IGF2BP2

Insulin‐like growth factor 2 mRNA‐binding protein 2 (IGF2BP2) has been found to bind not only insulin‐like growth factor 2 (IGF2) mRNA but also various mRNAs and lncRNAs. In this section, we introduce 10 recently reported cases of IGF2BP2 binding. IGF2BP2 binds to METTL14 m^6^A‐modified TGFB2 mRNA and upregulates TGFB2 expression, resulting in enhanced gemcitabine resistance in pancreatic cancer. Mechanistically, TGFB2 upregulates the expression of lipogenesis regulator sterol regulatory element binding factor 1 (SREBF1) and downstream lipogenic enzymes via PI3K/AKT signaling [28]. One of the methyltransferases, vir‐like m^6^A methyltransferase associated (VIRMA), is highly expressed in pancreatic cancer and modifies the 3’ UTR of signaling receptor and transporter of retinol STRA6 (STRA6) mRNA with m^6^A. The m^6^A‐modified STRA6 mRNA is stabilized by the binding of IGF2BP2, which increases downstream hypoxia‐inducible factor 1 subunit alpha (HIF‐1α) expression and enhances glycolysis [29]. High mobility group at‐hook 2 (HMGA2) is upregulated in pancreatic cancer. HMGA2 upregulates IGF2BP2 expression, stabilizes m^6^A‐modified amyloid beta precursor‐like protein 2 (APLP2) mRNA, and promotes pancreatic cancer. Knockdown of HMGA2 or IGF2BP2 in a mouse xenograft model suppressed cancer growth [30]. High expression of IGF2BP2 stabilizes m^6^A‐modified PD‐L1 mRNA, which increases PD‐L1 expression and promotes cancer growth [31]. Myosin 1C (MYO1C) mRNA is m^6^A‐modified by METTL3 and generates circMYO1C by back splicing. CircMYO1 is upregulated in pancreatic cancer and stabilizes m^6^A‐modified PD‐L1 mRNA with IGF2BP2 to upregulate PD‐L1 expression, promoting immune escape [32]. Cleavage stimulation factor subunit 2 (CSTF2), which is involved in posttranscriptional regulation, assists the m^6^A modification of METTL3 by slowing the RNA Pol II elongation rate during the transcription of target genes. CSTF2‐mediated m^6^A modification is mainly recognized by IGF2BP2, and centromere protein F (CENPF), wnt family member 7b (WNT7B), and neurotensin receptor 1 (NTSR1) were confirmed to be targets of m^6^A modification by CSTF2 [33]. TCGA and Gene Expression Omnibus (GEO) database analysis revealed that IGF2BP2 is involved in the prognosis of pancreatic cancer and is a pancreatic cancer marker. UDP‐GlcNAc:betaGal beta‐1,3‐*N*‐acetylglucosaminyltransferase 6 (B3GNT6) mRNA is modified with m^6^A, which is stabilized by IGF2BP2 binding and contributes to pancreatic cancer progression [34]. Polo‐like kinase 1 (PLK1) mRNA is modified by m^6^A and stabilized by binding to IGF2BP2, which increases PLK1 expression and contributes to cancer progression. Suppression of PLK1 by m^6^A removal of PLK1 mRNA causes replicating stress and mitotic catastrophe, induces apoptosis by activating the Rad3‐related (ATR) signaling pathway [35]. IGF2BP2 was reported to bind to lncRNA as well as mRNA. LINC00941 is m^6^A‐modified by METTL14 and stabilized by IGF2BP2 binding, contributing to the migration and invasion of cancer cells [36]. lncRNA‐PACERR is highly expressed in tumor‐associated macrophages (TAMs) of pancreatic cancer and increases M2‐polarized cells. lncRNA‐PACERR is m^6^A‐modified and stabilized by IGF2BP2, which activates the KLF12/p‐AKT/c‐Myc pathway by binding to miR‐671‐3p [37]. Thus, IGF2BP2 was found to be a positive regulator of cancer growth.

### 3.2 IGF2BP1 and IGF2BP3

New reports have also been made on insulin‐like growth factor 2 mRNA‐binding protein 1 (IGF2BP1) and insulin‐like growth factor 2 mRNA‐binding protein 3 (IGF2BP3). miR‐383‐5p is modified by m^6^A and stabilized by IGF2BP1, preventing its destabilization by FTO alpha‐ketoglutarate‐dependent dioxygenase (FTO). However, in pancreatic cancer, downregulation of miR‐383‐5p by upregulation of FTO promotes cancer progression by upregulating the expression of integrin subunit alpha 3 (ITGA3), a target of miR‐383‐5p [38]. Glucan branching enzyme 1 (GBE1), which is involved in cellular glycogen metabolism, is upregulated in pancreatic cancer and is associated with poor prognosis. Overexpression of GBE1 promotes the proliferation of pancreatic cancer cells, whereas knockdown of GBE1 alleviates the malignant phenotype. wt1‐associated protein (WTAP)/IGF2BP3 is the m^6^A regulator of GBE1, and m^6^A modification of GBE1 mRNA upregulates GBE1 expression, resulting in the upregulation of downstream c‐Myc expression [39]. Spermine synthase (SMS) mRNA is m^6^A‐modified by METTL3. IGF2BP3 stabilizes SMS mRNA and upregulates SMS. SMS is upregulated in pancreatic cancer and suppresses spermidine accumulation by converting spermidine to spermine, activating AKT and the epithelial‐mesenchymal transition (EMT) signaling pathway [40]. Thus, IGF2BP1 and IGF2BP3 were also found to be involved in gene expression related to cancer progression. Although most of them are cancer‐promoting, future exploration of cases in which they act in a cancer‐suppressive manner is also expected.

### 3.3 YTHDF1

This section presents four recent reports on yth n6‐methyladenosine RNA‐binding protein f1 (YTHDF1). F‐box protein 31 (FBXO31) is upregulated in pancreatic cancer and worsens the prognosis. m^6^A modification by METTL3 stabilizes FBXO31 mRNA by binding to YTHDF1, which increases FBXO31 expression and promotes proteasome‐dependent degradation of sirtuin 2 (SIRT2), leading to cancer progression [41]. Dead‐box helicase 23 (DDX23) mRNA is m^6^A‐modified by METTL3 and is stabilized by YTHDF1, upregulating DDX3. DDX23 activates AKT signaling and promotes cancer progression [42]. Phd finger protein 10 (PHF10) mRNA is m^6^A‐modified by zinc finger ccch‐type containing 13 (ZC3H13) and stabilized by YTHDF1 binding. PHF10 is a member of the PBAF chromatin‐remodeling complex. When PHF10 expression is downregulated by fisetin, a DNA damage‐inducing anticancer drug, DNA damage accumulates, and homologous recombination (HR) repair is decreased [43]. LINC00901 is m^6^A‐modified and destabilized by YTHDF1 binding. When LINC00901 is upregulated, IGF2BP2 is also upregulated, stabilizing MYC mRNA, which in turn upregulates MYC and promotes cancer progression [44]. Thus, mRNA may be destabilized as well as stabilized by YTHDF1 binding. Further mechanistic clarification may be needed to determine the cause of the destabilization.

### 3.4 YTHDF2

This section presents five recent reports on yth N6‐methyladenosine RNA‐binding protein F2 (YTHDF2). Inhibitor of DNA binding 2 (ID2) mRNA is modified with m^6^A by METTL3 and stabilized by YTHDF2, resulting in increased expression of ID2. ID2 induces the expression of nanog homeobox (NANOG) and sry‐box transcription factor 2 (SOX2), which are cancer stem cell markers, through the PI3K/AKT pathway, and contributes to cell proliferation and cancer stem cell maintenance [45]. Histone deacetylase type 4 (HDAC4) mRNA is stabilized and upregulated by m^6^A modification and binding to YTHDF2, which enhances HIF1a expression. Increased expression of HIF1a in hypoxia promotes cancer growth by activating glycolysis [46]. Cugbp elav‐like family member 2 (CELF2), an alternative splicing regulator, is stabilized by alkb homolog 5, RNA demethylase (ALKBH5) through regulation of m^6^A modification of mRNA, but when ALKBH5 expression is decreased, YTHDF2 binds to the m^6^A modification site and promotes its degradation. As a result, alternative splicing from CD44s to CD44V is suppressed, CD44s expression increases, and the endoplasmic reticulum‐associated degradation (ERAD) signaling pathway is activated, leading to cancer progression [47]. Period circadian regulator 1 (PER1) mRNA m^6^A modification by METTL3/METTL14 promoted mRNA degradation by binding to YTHDF2, but removal of m^6^A by ALKBH5 increased PER1 expression, activating ATM‐CHK2‐P53/CDC25C signaling [48]. YTHDF2 is upregulated in pancreatic cancer. YTHDF2 suppresses yes1‐associated transcriptional regulator (YAP) expression and contributes to the activation of TGFβ/Smad signaling [49]. Thus, YTHDF2 binding can stabilize or destabilize mRNA. Further studies are needed to determine what factors determine this.

In addition, the regulation of gene expression by YTHDF2 seems to be cooperative with the eraser, ALKBH5. It will be interesting to see when YTHDF2 or ALKBH5 becomes dominant.

### 3.5 YTHDF3

This section presents two recent reports on YTHDF3. DICER1‐AS1 is downregulated in pancreatic cancer, and its expression is negatively correlated with glycolytic genes expression. DICER1‐AS1 is degraded by YTHDF3 binding upon m^6^A modification. DICER1‐AS1 promotes DICER1 expression by recruiting YY1 to the promoter of DICER1. miR‐5586‐5p processed by DICER1 binds to YTHDF3 mRNA and represses YTHDF3 expression. miR‐5586‐5p also suppresses the expression of glycolytic genes, lactate dehydrogenase A (LDHA), hexokinase 2 (HK2), phosphoglycerate kinase 1 (PGK1), and solute carrier family 2 member 1 (SLC2A1), thereby suppressing glycolysis and cancer progression [50]. In pancreatic cancer with proteasome inhibition by celastrol, METTL3 expression is decreased, m^6^A levels of Claspin and Bcl‐2 mRNA are lowered, and their expressions are suppressed by YTHDF3 binding, resulting in suppression of cell growth [51]. Thus, YTHDF3 destabilizes mRNA and suppresses gene expression. As described above, readers regulate the expression of various genes.

### 3.6 Other Readers

**FIGURE 3:**
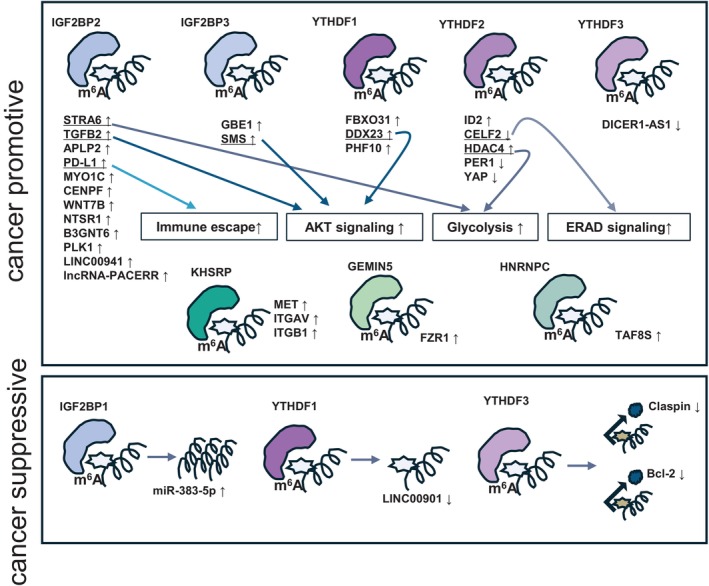
Regulation of gene expression by readers. Binding of readers increases or decreases gene expression.

This section presents three recent reports on readers other than IGF2BP family and YTHDF family. KH‐type splicing regulatory protein (KHSRP) binds to and stabilizes m^6^A of the met proto‐oncogene, receptor tyrosine kinase (MET), integrin subunit alpha v (ITGAV), and integrin subunit beta 1 (ITGB1) mRNAs, activating the FAK signaling pathway and promoting pancreatic cancer growth [52]. Fizzy and cell division cycle 20 related 1 (FZR1) mRNA is m^6^A‐modified, and the binding of gem nuclear organelle‐associated protein 5 (GEMIN5) to the m^6^A‐modified site recruits eukaryotic translation initiation factor 3 subunit a (eIF3) which upregulates FZR1 expression, maintaining the G0–G1 quiescent state and enhancing gemcitabine resistance [53]. High expression of heterogeneous nuclear ribonucleoprotein C (HNRNPC) in pancreatic cancer is associated with metastasis and poor prognosis. TATA‐box binding protein‐associated factor 8 (TAF8) has an m^6^A modification site at exon 7, and if this site is not m^6^A‐modified, splicing of TAF8L, an antimetastatic isoform, occurs, but if m^6^A is modified, HNRNPC binds to the site, and TAF8S, a pro‐metastatic alternative splicing isoform, is generated by alternative splicing, which leads to progression of metastasis [54]. Thus, various readers are involved in the regulation of gene expression of cancer‐related genes (Figure 3 and Table 2).

**TABLE 2:** Targets of readers and their functions in pancreatic cancer.

Readers	Targets	Expression	Binding effect on tumor	Experiments	References
IGF2BP2	TGFB2	Up	Promotive	Cell lines and mouse	[28]
IGF2BP2	STRA6	Up	Promotive	Cell lines, mouse, and human PC tissues	[29]
IGF2BP2	APLP2	Up	Promotive	Cell lines and mouse	[30]
IGF2BP2	PD‐L1	Up	Promotive	Cell lines and mouse	[31]
IGF2BP2	MYO1C	Up	Promotive	Cell lines and mouse	[32]
IGF2BP2	CENPF, WNT7B, and NTSR1	Up	Promotive	Cell lines and mouse	[33]
IGF2BP2	B3GNT6	Up	Promotive	Cell lines and mouse	[34]
IGF2BP2	PLK1	Up	Promotive	Cell lines, mouse, and human PC tissues	[35]
IGF2BP2	LINC00941	Up	Promotive	Cell lines, mouse, and human PC tissues	[36]
IGF2BP2	lncRNA‐PACERR	Up	Promotive	Cell lines, mouse, and human PC tissues	[37]
IGF2BP1	miR‐383‐5p	Up	Suppressive	Cell lines	[38]
IGF2BP3	GBE1	Up	Promotive	Cell lines and mouse	[39]
IGF2BP3	SMS	Up	Promotive	Cell lines, mouse, and human PC tissues	[40]
YTHDF1	FBXO31	Up	Promotive	Cell lines, mouse, and human PC tissues	[41]
YTHDF1	DDX23	Up	Promotive	Cell lines, mouse, and human PC tissues	[42]
YTHDF1	PHF10	Up	Promotive	Cell lines	[43]
YTHDF1	LINC00901	Down	Suppressive	Cell lines and mouse	[44]
YTHDF2	ID2	Up	Promotive	Cell lines, mouse, and human PC tissues	[45]
YTHDF2	HDAC4	Up	Promotive	Cell lines	[46]
YTHDF2	CELF2	Down	Promotive	Cell lines and mouse	[47]
YTHDF2	PER1	Down	Promotive	Cell lines, mouse, and human PC tissues	[48]
YTHDF2	YAP	Down	Promotive	Cell lines	[49]
YTHDF3	DICER1‐AS1	Down	Promotive	Cell lines and mouse	[50]
YTHDF3	Claspin and Bcl‐2	Down	Suppressive	Cell lines and mouse	[51]
KHSRP	MET, ITGAV, and ITGB1	Up	Promotive	Cell lines and mouse	[52]
GEMIN5	FZR1	Up	Promotive	Cell lines and mouse	[53]
HNRNPC	TAF8	Up (TAF8S)	Promotive	Cell lines, mouse, and human PC tissues	[54]

Abbreviation: PC, pancreatic cancer.

## 4 Erasers

### 4.1 ALKBH5

This section presents seven recent reports on ALKBH5. Analysis of gene expression and copy number variation (CNV) data of pancreatic cancer patients in the TCGA database reported that patients with ALKBH5 CNV have a worse prognosis and that increased expression of ALKBH5 is positively correlated with activation of AKT pathways [55]. The expression of f‐box and leucine‐rich repeat protein 5 (FBXL5), a ubiquitin ligase, is regulated by m^6^A modification of mRNA and is upregulated by m^6^A removal of ALKBH5. FBXL5 suppresses cancer progression by decreasing intracellular iron pools through ubiquitination of iron‐responsive element binding protein 2 (IRP2), an iron‐regulatory protein [56]. ALKBH5 expression is downregulated by gemcitabine treatment, but overexpression increases gemcitabine sensitivity. ALKBH5 upregulates Wnt inhibitory factor 1 (WIF‐1) expression by removing m^6^A of WIF‐1 mRNA and inhibits pancreatic cancer progression by suppressing the Wnt pathway [57]. ALKBH5 is downregulated in pancreatic cancer. KCNK15‐AS1 is modified with m^6^A and destabilized, but KCNK15‐AS1 is upregulated when m^6^A is removed by ALKBH5 and suppresses migration and invasion [58]. In addition, KCNK15‐AS1 upregulation recruits MDM2 to activate phosphatase and tensin homolog (PTEN) transcription by promoting the ubiquitination of RE1 silencing transcription factor (REST), and PTEN expression suppresses the AKT pathway and inhibits cancer progression [59]. DDIT4‐AS1 is significantly upregulated in pancreatic cancer, and its elevated expression is correlated with poor prognosis. DDIT4‐AS is modified with m^6^A and stabilized by binding of elav‐like RNA‐binding protein 1 (HuR), which inhibits the binding of SMG5 nonsense‐mediated mRNA decay factor (SMG5) and protein phosphatase 2a (PP2A) to UPF1 RNA helicase and ATPase (UPF1), thereby promoting UPF1 phosphorylation, which has the ability to degrade mRNA, and DNA damage‐inducible transcript 4 (DDIT4) mRNA degradation. mTOR pathway is then activated, increasing stemness and gemcitabine resistance. ALKBH5 removes DDIT4‐AS m^6^A and suppresses DDIT4‐AS expression [60]. ALKBH5 is upregulated in pancreatic neuroendocrine neoplasms (pNENs). Fatty acid binding protein 5 (FABP5) mRNA is m^6^A‐modified, but upregulation of ALKBH5 increases FABP5 expression by decreasing m^6^A modification, inducing fatty acid synthesis via PI3K/AKT/mTOR signaling pathway and promoting cancer growth [61]. Thus, although many reports indicate that ALKBH5 functions in a cancer‐suppressive manner, it has also been reported to be cancer‐promoting in some cancer types. There may be some special factor that causes the cancer to grow despite the elevated expression of ALKBH5.

### 4.2 FTO

**FIGURE 4:**
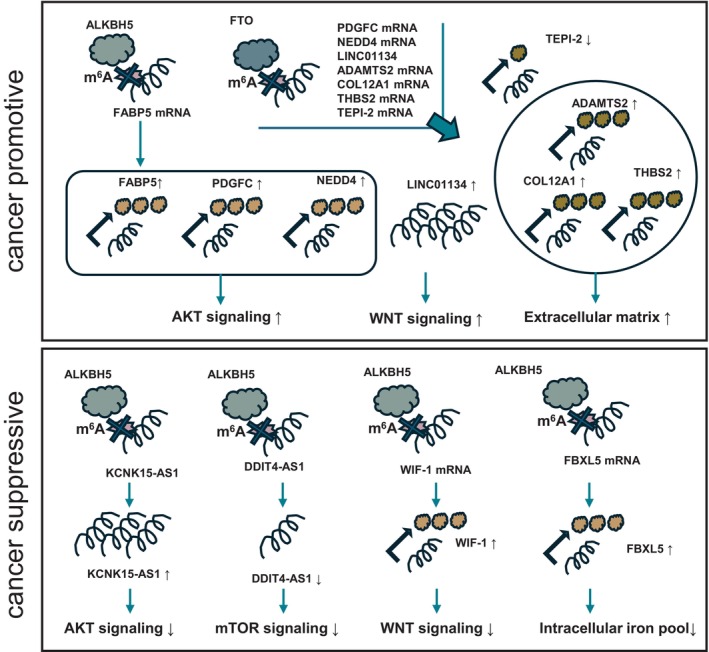
Regulation of gene expression by erasers. Removal of RNA modifications by erasers increases or decreases gene expression.

This section presents five recent reports on FTO. FTO is highly expressed in pancreatic cancer and is associated with poor prognosis. Knockdown of FTO suppressed cancer growth in a mouse xenograft model by increasing the m^6^A level of platelet‐derived growth factor C (PDGFC) mRNA, promoting PDGFC mRNA degradation by binding to YTHDF2, decreasing PDGFC secretion, and inhibiting the AKT signaling pathway [62]. Tissue factor pathway inhibitor 2 (TFPI‐2) mRNA is m^6^A‐modified and stabilized by binding to YTHDF1, which upregulates TFPI‐2 expression and suppresses tumor growth, migration, and invasion, but removal of m^6^A‐modification by FTO reduces TFPI‐2 expression and promotes cancer progression [63]. NEDD4 E3 ubiquitin protein ligase (NEDD4) mRNA is stabilized by YTHDF2 in a low m^6^A‐modified state, and NEDD4 enhances gemcitabine resistance by activating the PI3K/AKT pathway. Knockdown of FTO increases gemcitabine sensitivity by suppressing NEDD4 expression and increasing PTEN expression [64]. ADAM metallopeptidase with thrombospondin type 1 motif 2 (ADAMTS2), collagen type xii alpha 1 chain (COL12A1), and thrombospondin 2 (THBS2), which contribute to extracellular matrix (ECM) formation, are regulated by m^6^A modification of mRNA, and knockdown of FTO reduces their mRNA levels and m^6^A modification levels, suppressing migration and invasion of pancreatic cancer cells [65]. LINC01134 is upregulated in pancreatic cancer and enhances gemcitabine resistance by promoting stem cell features and regulating the cell cycle. LINC01134 sponges the tumor suppressor gene miR‐497‐5p and upregulates Wnt family member 5A (WNT5A) expression. Binding of YTHDF2 to m^6^A‐modified LINC01134 suppresses LINC01134 expression, which is upregulated when m^6^A is removed by FTO [66]. Thus, FTO has been reported to be an eraser that functions in a cancer‐promoting manner. As described above, erasers regulate the expression of various genes but may tend to be bifurcated into cancer‐promoting and cancer‐suppressing (Figure 4 and Table 3).

**TABLE 3:** Targets of erasers and their functions in pancreatic cancer.

Erasers	Targets	Expression	Erasing effect on tumor	Experiments	References
ALKBH5	FBXL5	Up	Suppressive	Cell lines and human PC tissues	[56]
ALKBH5	WIF‐1	Up	Suppressive	Cell lines and human PC tissues	[57]
ALKBH5	KCNK15‐AS1	Up	Suppressive	Cell lines	[58, 59]
ALKBH5	DDIT4‐AS1	Down	Suppressive	Cell lines and mouse	[60]
ALKBH5	FABP5	Up	Promotive	Cell lines and mouse	[61]
FTO	PDGFC	Up	Promotive	Cell lines and human PC tissues	[62]
FTO	TFPI‐2	Down	Promotive	Cell lines and human PC tissues	[63]
FTO	NEDD4	Up	Promotive	Cell lines and mouse	[64]
FTO	ADAMTS2, COL12A1 and THBS2	Up	Promotive	Cell lines	[65]
FTO	LINC01134	Up	Promotive	Cell lines and human PC tissues	[66]

Abbreviation: PC, pancreatic cancer.

## 5 m^6^A MeRIP‐Seq of Pancreatic Cancer Tissues

We recently published our findings on m6A markers with potential diagnostic utility for pancreatic cancer [67]. The top 20 genes with elevated m^6^A modification in tumors compared to normal tissue ducts and acinars were insulin (INS), trefoil factor 1 (TFF1), zinc finger c2hc‐type containing 1b (ZC2HC1B), claudin 2 (CLDN2), RMDN2 antisense RNA 1 (RMDN2‐AS), proteasome 20s subunit alpha 6 (PSMA6), LINC00396, hemopexin (HPX), LOC101928324, MIR 4791, LOC100288842, insulin‐like 4 (INSL4), CD14, neuropeptide s receptor 1 (NPSR1), transcription elongation factor a Like 8 (TCEAL8), lipocalin 2 (LCN2), amylase alpha 2a (AMY2A), spanx family member n2 (SPANXN2), thrombospondin type 1 domain containing 1 (THSD1), and APCDD1L‐AS1. Gene set enrichment analysis (GSEA) revealed that genes with elevated m^6^A modifications in the tumors were involved in glycan biosynthesis, complement and coagulation cascades, systemic lupus erythematosus, TGF beta signaling pathway, ECM receptor interaction, and leukocyte transendothelial migration. The top 20 genes with elevated m^6^A modification in tumors were confirmed in the pancreatic cancer single‐cell RNA‐sequencing (scRNA‐seq) database we previously constructed, and expression was confirmed in eight genes: INS, TFF1, CLDN2, PSMA6, CD14, TCEAL8, LCN2, and AMY2A. Although the function of TCEAL8 in cancer was unknown, we found that TCEAL8 expression activated oxidative phosphorylation, protein export, ubiquitin mediated proteolysis, fatty acid metabolism, and antigen presentation through visium analysis of cancer tissues. Therefore, further functional analysis is expected in the future.

## 6 Other RNA Modifications

In addition to m^6^A, other RNA modifications that affect gene expression have been reported. Methyltransferase 8 (METTL8) methylates the C32 site of mitochondrial proteins mt‐tRNASer (UCN) and mt‐tRNAThr, resulting in m^3^C. METTL8 is upregulated in pancreatic cancer and is associated with poor prognosis. Knockdown of METTL8 decreases respiratory chain activity, suggesting that m^3^C modification of mt‐tRNA activates respiratory chain activity [68]. Nuclear cap binding protein subunit 2 (NCBP2), an m^7^G‐binding protein, is upregulated in pancreatic cancer and is associated with poor prognosis. Knockdown of NCBP2 suppressed the growth of pancreatic cancer, while overexpression of NCBP2 promoted the growth of pancreatic cancer. NCBP2 binds to m^7^G‐modified c‐JUN mRNA and enhances MEK/ERK signaling by upregulating c‐JUN translation, thereby promoting pancreatic cancer growth [69]. m^1^A has been detected in tRNA, ribosomal RNA, and mRNA and has been reported to be involved in stabilizing RNA structure and regulating gene expression, but the details of the function of m^1^A modification in each gene are still unclear [70]. Interesting reports on the functions of 5mC and ac4C have been published, although not in pancreatic cancer. Aly/REF export factor (ALYREF) is upregulated in hepatocellular carcinoma and is associated with poor prognosis. ALYREF binds to m^5^C‐modified epidermal growth factor receptor (EGFR) mRNA and promotes cancer growth by stabilizing EGFR mRNA, upregulating EGFR expression and enhancing the STAT3 signaling pathway [71]. *N*‐acetyltransferase 10 (NAT10) is upregulated in hepatocellular carcinoma and is associated with poor prognosis. High mobility group box 2 (HMGB2) mRNA is ac^4^C‐modified by NAT10, and binding of eukaryotic translation elongation factor 2 (EEF2) to NAT10 increases HMGB2 expression and promotes cancer proliferation [72]. Thus, most of the functions of RNA modifications other than m^6^A are still unknown in pancreatic cancer. Therefore, more and more research is needed in the future.

## 7 Conclusions

As described above, m^6^A regulates the expression of genes involved in important signals for cancer growth in pancreatic cancer. The development of inhibitors targeting writers and erasers is currently underway. However, since writers and erasers target many genes, such inhibitors may have a broad impact on gene expression. The functions of m^6^A in individual genes have been elucidated one by one. Based on this information, we hope that controlling individual m^6^A modifications will facilitate the treatment of pancreatic cancer. As the technology to detect modifications at specific sites of RNA becomes more advanced, m^6^A‐modified RNAs may serve as potential biomarkers to support the early detection of pancreatic cancer. Although this review describes the functions of RNA modifications in pancreatic cancer, these functions may not be universally applicable to other cancer types. For instance, while ALKBH5 exhibits tumor‐suppressive activity in pancreatic cancer, it plays a tumor‐promoting role in glioblastoma [73]. Therefore, further functional investigations specific to each cancer type are warranted to fully understand the context‐dependent roles of RNA modifications in tumorigenesis. With that in mind, further research is still needed for the social implementation of cancer diagnosis and therapeutics using m^6^A modification data.

## Author Contributions

H.I., T.H., and T.K. contributed to conceptualization. T.H., S.M., Y.A., Y.S., and K.I. collected references and made tables. T.H., S.M., K.O., and H.I. wrote the manuscript. All authors contributed to review the manuscript.

## Ethics Statement

The authors have nothing to report.

## Conflicts of Interest

Partial institutional endowments were received from Hirotsu Bio Science Inc. (Tokyo, Japan), Kinshu‐kai Medical Corporation (Osaka, Japan), Kyowa‐kai Medical Corporation (Kawanishi, Hyogo and Osaka, Japan), IDEA Consultants Inc. (Tokyo, Japan), and Unitech Co. Ltd. (Chiba, Japan). K.O. is an employee of IDEA Consultants Inc. T. K. is CEO of Kyowa‐kai Medical Corporation. H.I. is the associate editors of this journal. Others have no conflicts of interest for this review.

## Data Availability

No new data have been taken in this review.
